# Functional Connectivity with the Default Mode Network Is Altered in Fibromyalgia Patients

**DOI:** 10.1371/journal.pone.0159198

**Published:** 2016-07-21

**Authors:** Nicholas Fallon, Yee Chiu, Turo Nurmikko, Andrej Stancak

**Affiliations:** 1 Department of Psychological Sciences, Institute of Psychology, Health, and Society, University of Liverpool, Liverpool, United Kingdom; 2 Wirral University Teaching Hospital NHS Foundation Trust, Wirral, United Kingdom; 3 Pain Research Institute, Institute of Ageing and Chronic Disease, University of Liverpool, Liverpool, United Kingdom; 4 The Walton Centre NHS Foundation Trust, Liverpool, United Kingdom; University of Texas at Austin, UNITED STATES

## Abstract

Fibromyalgia syndrome (FMS) patients show altered connectivity with the network maintaining ongoing resting brain activity, known as the default mode network (DMN). The connectivity patterns of DMN with the rest of the brain in FMS patients are poorly understood. This study employed seed-based functional connectivity analysis to investigate resting-state functional connectivity with DMN structures in FMS. Sixteen female FMS patients and 15 age-matched, healthy control subjects underwent T2-weighted resting-state MRI scanning and functional connectivity analyses using DMN network seed regions. FMS patients demonstrated alterations to connectivity between DMN structures and anterior midcingulate cortex, right parahippocampal gyrus, left superior parietal lobule and left inferior temporal gyrus. Correlation analysis showed that reduced functional connectivity between the DMN and the right parahippocampal gyrus was associated with longer duration of symptoms in FMS patients, whereas augmented connectivity between the anterior midcingulate and posterior cingulate cortices was associated with tenderness and depression scores. Our findings demonstrate alterations to functional connectivity between DMN regions and a variety of regions which are important for pain, cognitive and emotional processing in FMS patients, and which may contribute to the development or maintenance of chronic symptoms in FMS.

## Introduction

Fibromyalgia syndrome is characterised by widespread chronic pain, cognitive and affective disturbances and fatigue. The pathophysiology of FMS is unknown with evidence for both central and peripheral contributions [1], however, ongoing chronic pain was previously shown to cause reorganisation of the default mode network (DMN) in the brain [2], and resting-state functional connectivity changes are prevalent in FMS (reviewed in [3]). Furthermore, functional connectivity alterations can reflect the transition from acute to chronic pain [4]. Altered central connectivity could contribute to the development and maintenance of fibromyalgia or reflect predominant symptoms in patients, and improved understanding of the mechanisms underlying FMS symptoms could aid development of therapeutic and diagnostic interventions.

The DMN comprises of regions which demonstrate coherent activation patterns [5,6] and functional connectivity [7] when the brain switches between rest and task states. DMN regions include precuneus, posterior cingulate cortex (PCC), bilateral inferior parietal cortices and medial prefrontal cortices (mPFC) [8–10]. Functional connectivity with the DMN network can be quantitatively evaluated using correlational analysis of resting-state fMRI data, utilising either a hypothesis led seed based approach with known DMN regions of interest [8,11,12], or data-driven independent component analysis (ICA) to identify resting-state networks [13]. ICA of FMS patients has indicated enhanced intrinsic connectivity between the DMN and insula cortices [14], which was shown to subside following a non-pharmacological intervention [15]. Seed based research demonstrated augmented connections between somatosensory cortices and DMN in FMS patients [16], and altered connectivity between pain processing structures such as anterior cingulate cortex (ACC) and insula cortex, or mPFC with PCC [17]. Similarly, augmented mPFC-PCC connections have been reported in other chronic pain states such as temporomandibular disorder [18], and ACC-PCC connectivity was enhanced in chronic back pain patients [19]. Research utilising seeds located in insular cortices revealed increased functional connectivity with cingulate cortex in FMS patients [20], although another study did not identify augmented insula-DMN connectivity, but indicated that insula-somatosensory cortex connectivity was affected by FMS [21].

Disruption of the DMN could have significant implications for cognitive and behavioural impairments in chronic pain [2], and recent studies have indicated that various successful therapeutic interventions in FMS patients correspond with normalisation of a wide range of resting-state functional connectivity alterations [15,21–23]. Therefore, functional connectivity analysis of resting-state networks in FMS patients not only has potential to enhance our understanding of mechanisms underlying symptoms, but could also be used to improve clinical interventions or diagnosis. Unfortunately, it appears that alterations to DMN connectivity patterns vary across different studies of FMS patients, which reflects the variability seen in structural brain changes (reviewed in [24]). As brain imaging studies typically employ small cohorts of patients, further data is needed to allow for the possibility of a future meta-analytic evaluation of the strongest and most relevant functional connectivity changes in FMS patients.

We utilised seed-based functional connectivity analysis to evaluate resting-state functional connectivity with DMN structures selected from meta-analyses [9], and clinical measures including duration of symptoms (years), manual tender point scores (MTPS, [25]), and Beck Depression Inventory (BDI, [26]) scores. We hypothesised that the DMN would show functional connectivity alterations in FMS patients with structures relevant to FMS symptoms such as insula and cingulate cortices for pain processing, somatosensory cortices, or pre-frontal/limbic regions important for cognitive and affective processing.

## Methods

### Participants

Sixteen female patients (age 38.5 ± 8.45 years, mean ± SD) took part in the study. Mean duration of symptoms was 9.13 ± 6.80 years, and mean time since diagnosis was 2.88 ± 1.34 years (mean ± SD). All patients fulfilled ACR criteria for diagnosis with fibromyalgia on the day of scanning [25]. The recruitment, medication and symptom profiles for the patient population are reported in our previous morphological report [27]. Five patients were using no medications for management of their FMS and the remaining 11 patients were either using permissible doses of common medications with minimal central nervous efficacy or withdrew from non-permitted medications, such as co-codamol, for at least 3 days prior to recordings. Informed written consent was obtained from all participants in accordance with the Declaration of Helsinki and the study was approved by the Research Ethics Committee of the University of Liverpool and the Research Governance Committee of two NHS Foundation Trust hospitals; the Walton Centre, Liverpool, United Kingdom, and Wirral University Teaching Hospital, Wirral, United Kingdom. Fifteen age-matched female controls (age 39.40 ± 8.65 years, mean ± SD) were recruited through campus advertisement. Volunteers taking regular medication, currently diagnosed with any disease or disorder or demonstrating a history of major disease, alcohol/drug abuse or serious head or brain injury were excluded. Patients and volunteers were compensated for time and travel expenses. Table 1 shows demographic and clinical data for healthy and patient groups, and the results of T-tests to compare mean scores on clinical measures for each group.

**Table 1:** FMS patient and healthy control clinical and demographic data FIQ = Fibromyalgia Impact Questionnaire; MTPS = Manual Tender Point Scale; BDI = Beck Depression Inventory; SD = Standard deviation.

	FMS		Healthy			
	Mean ± SD		Mean ± SD		T	*P*
Age	38.45	8.45	39.40	8.65	-0.14	0.89
FIQ	62.37	15.84	6.26	6.51	12.74	<0.001
MTPS	4.66	1.94	0.26	0.34	8.67	<0.001
BDI	19.5	11.19	3.87	4.42	5.05	<0.001
Years symptoms	9.13	6.80	0.00	0.0	5.19	<0.001

### MRI data acquisition

Magnetic resonance images were acquired using a whole-body 3 tesla Siemens Trio MRI imaging system (Siemens, Magnetom, Erlangen, Germany) and an 8-channel head coil. Resting-state fMRI data were acquired using a T2-weighted sequence (32 axial slices, 0.7mm spacing, TR = 2.0 s, TE = 30 ms, flip angle = 90°, field of view = 192mm, voxel size = 3× 3 ×3.5 mm). During a 20 minute resting-state fMRI acquisition period (600 scans), participants were asked to remain awake with their eyes closed. Fifteen auditory stimuli (a one second beep tone) were delivered via headphones at pseudorandom intervals (every 60–90 s, mean onset 75 s). Participants were instructed to respond to the auditory stimulus by pressing a button on a button box placed in their right hand. Stimulus-response epochs were later defined as blocks and excluded from the analysis to leave interleaved resting-state data.

### Pre-processing and data analysis

Spatial pre-processing was performed in SPM8 (Welcome Trust Centre for Neuroimaging, University College London, United Kingdom) running in Matlab v.7.13 (The Mathworks Inc, USA). Functional volumes underwent realignment, slice-timing correction, normalisation to Montreal Neuroloigical Institute (MNI) space using the normalised EPI template image in SPM and spatial smoothing (8mm full width half maximum Gaussian kernel filter). Motion parameters from realignment were evaluated and a motion artefact threshold (translation > 3mm, rotation > 1 degree) was employed for exclusion. No participants displayed gross movements to require exclusion. Noise correction was performed using the anatomical component-based noise correction (aCompCor) method [28] implemented in the Functional Connectivity Toolbox (CONN, [11]) in SPM8. During pre-processing, high-resolution T1-weighted anatomical volumes for each subject were segmented into grey matter, white matter and cerebrospinal fluid and normalised to MNI space. BOLD signals from cerebral white matter and ventricles were removed using principal component analysis (PCA) of the multivariate BOLD signal within each these masks [11]. The residual BOLD data was previously shown to benefit from improved specificity, sensitivity and validity for subsequent functional connectivity analyses [11,29]. Sound conditions, representing the 15 second period beginning 1 second before onset of a sound stimuli/response epoch and 14 seconds post stimuli, were defined as blocks and removed from the data so as to only investigate the remaining interleaved resting-state data. This method was previously shown to provide appropriate data for resting-state network analysis [30]. Finally, BOLD data was bandpass filtered (0.008–0.09 Hz) to reduce low-frequency drift and noise effects.

### Seed regions of interest

ROI seeds, consisting of 10mm diameter spheres, were defined in posterior cingulate cortex (PCC), precuneus (pC), medial prefrontal gyrus (MPFG), bilateral Inferior parietal lobules (L/R.IPL), right middle temporal gyrus (R.MTG) and ventral anterior cingulate cortex (vACC). These ROIs were centred on co-ordinates from a meta-analysis of DMN fMRI studies [9], and were generated using MarsBaR software (MRC Cognition and Brain Sciences Unit, Cambridge, United Kingdom) in SPM8. Table 2 shows the anatomical location, Brodmann area and MNI co-ordinates for each of the seed regions utilised. The BOLD time series extracted for each seed region was the average for all voxels making up the 10mm diameter sphere.

**Table 2:** Seed regions of interest selected for the default mode network; the seed location, hemisphere, Brodmann area and MNI (x,y,z) co-ordinates are shown network (adapted from [9]).

Hemisphere	Region	BA	*x*	*y*	*z*
Left	Precuneus (pC)	7	−4	−58	44
Left	Posterior cingulate (PCC)	31	−4	−52	22
Right	Ventral anterior cingulate (vACC)	32	2	32	−8
Right	Inferior parietal lobule (R.IPL)	40	52	−28	24
Left	Medial prefrontal cortex (mPFC)	9	−2	50	18
Right	Middle temporal gyrus (R.MTG)	39	46	−66	16
Left	Middle frontal gyrus (L.MFG)	8	−26	16	44
Left	Inferior parietal lobule (L.IPL)	40	−56	−36	28
Left	Middle temporal gyrus (L.MTG)	39	−42	−66	18

### Seed-to-voxel analysis

Individual correlation maps throughout the whole brain were generated in the CONN toolbox by extracting the mean resting-state BOLD time course from each seed ROI and calculating correlation coefficients with the BOLD timecourse of each voxel throughout the whole brain. The resulting coefficients were converted to normally distributed scores using Fisher's transformation to give maps of voxelwise functional connectivity for each seed ROI in the DMN for each subject. The value of each voxel throughout the whole brain represents the relative degree of functional connectivity with each seed [31]. These maps were subsequently used for second-level analysis of relative functional connectivity using a two-sided independent *t*-test, implemented in the CONN toolbox, to investigate differences in seed-to-voxel connectivity between groups. As in previous studies [32,33], voxelwise statistics throughout the whole brain were performed at an uncorrected level (P<0.001) before false discovery rate (FDR) correction [34] was applied at the cluster level (P<0.05).

### ROI-ROI Analyses

To examine whether functional connectivity between DMN structures differs in FMS, the average BOLD time series for all voxels in each seed ROI were normalised using Fisher’s transformation and correlations performed with all other seeds in the DMN network in a 9×9 correlation matrix. The resulting correlation coefficients for each participant were then compared using a two-sided independent samples *t*-test to evaluate between-group differences in ROI-ROI connectivity for each seed. Results were thresholded at P<0.05 and FDR correction was applied to correct for multiple tests required.

### Correlation analysis

Fischer transformed correlation coefficients for clusters of voxels showing a significant group difference in connectivity with DMN structures in seed-to-voxel analyses were extracted for each subject. One-tailed Pearson’s correlation analysis was performed to investigate the relationship between relative functional connectivity with seed ROIs, and clinical measures including duration of symptoms (years), tenderness scores measured on the day of scanning (MTPS), and Depression scores (BDI). As FMS patients demonstrated significantly higher scores on all clinical variables, with negligible scores evident in the healthy group, correlation analysis with clinical data was only performed within the patient population and a threshold (P<0.05) was employed to determine significance.

## Results

### Seed-to-voxel analysis

FMS patients, relative to healthy control participants, demonstrated significant connectivity differences between DMN seeds located in PCC, L.MFG and R.IPL and a variety of cortical regions. Table 3 shows the T-maxima of the clusters demonstrating altered connectivity with DMN structures, as well as MNI co-ordinates, P values (FDR corrected) and the size of each cluster in contiguous voxels.

**Table 3:** Seed-to-voxel analysis, brain regions showing alterations to functional connectivity with DMN seeds in FMS patients, relative to healthy control subjects. Cluster location, MNI co-ordinates (x,y,z) and T maxima (cluster-level FDR corrected) are shown, *k* = number of contiguous voxels. PCC = posterior cingulate cortex; L.MFG = left middle frontal gyrus; R.IPL = right inferior parietal lobule; ITG = inferior temporal gyrus; PHG = parahippocampal gyrus; aMCC = anterior midcingulate cortex; Hi = hippocampal formation; SPL = superior parietal lobule.

Seed	Contrast	Cluster	MNI [mm]	*k*	T	P
**PCC**	HC>FMS	ITG	60–30–12	283	-7.8	0.011
	HC>FMS	PHG	14, -12–24	264	-5.6	0.011
	FMS>HC	aMCC	-16, 18, 24	157	6.18	0.034
**R.IPL**	FMS>HC	Hi	26–14–14	335	6.91	0.034
**L.MFG**	FMS>HC	SPL	-32–62 50	294	4.92	0.024

**Fig 1:**
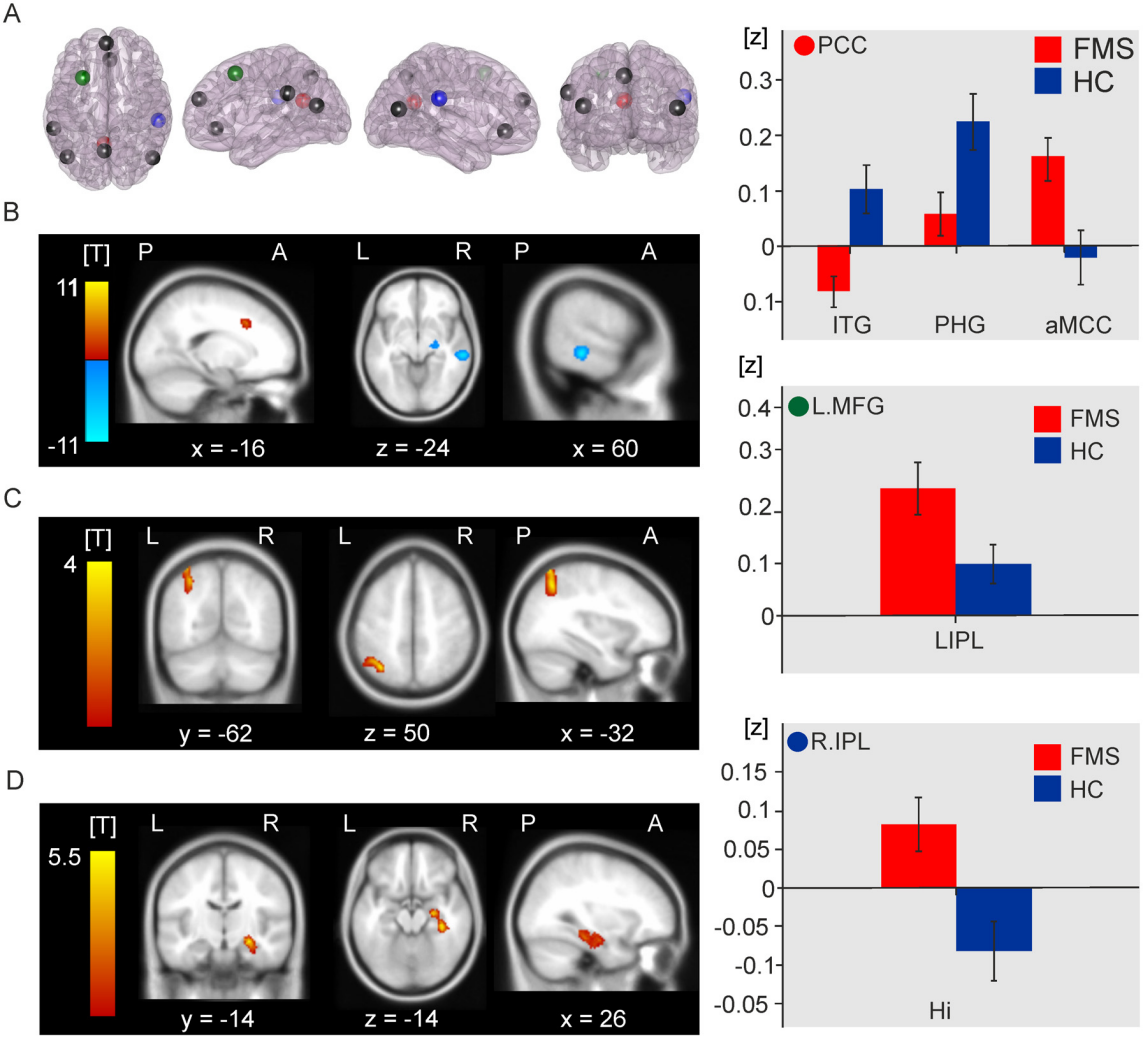
Seed–to-voxel analysis. **A.** The locations of all 9 DMN seeds as specified by [9]. The red sphere indicates the location of the PCC seed, green is the left MFG and blue for right IPL seed. **B.** Functional connectivity with the PCC seed. Red-yellow colour indicates a cluster showing increased connectivity with PCC in FMS patients relative to healthy participants; blue-white colour indicates clusters demonstrating reduced connectivity. *Top right panel;* mean Fischer transformed correlation coefficients indicating relative functional connectivity with PCC seed for each significant cluster in FMS and healthy groups. **C.** Functional connectivity with the left MFG seed. *Middle right panel;* mean Fischer transformed correlation coefficients indicating relative connectivity between left MFG seed and significant clusters in FMS and healthy control groups. **D.** Functional connectivity with the right IPL seed. *Bottom right panel;* mean Fischer transformed correlation coefficients indicating relative connectivity between right IPL seed and significant clusters in FMS and healthy control groups.

FMS patients exhibited reduced functional connectivity, relative to healthy controls, between the PCC seed in the DMN and the right parahippocampal gyrus (*t*(29) = -7.8, P = 0.011), and right inferior temporal gyrus (*t*(29) = −5.6, P = 0.011). FMS patients group also demonstrated enhanced connectivity, compared to healthy participants, between the right IPL seed and right hippocampal formation, (*t*(29) = 6.91, P = 0.034), the left MFG and left posterior parietal cortex (*t*(29) = 4.92, P = 0.024) and between the PCC seed and left anterior midcingulate cortex (aMCC, *t*(29) = 6.18, P = 0.034). Fig 1 shows the locations of DMN seeds (Fig 1A), as well as the locations of clusters of voxels demonstrating altered functional connectivity to DMN seeds in the FMS patient group relative to healthy control group, and histograms illustrating relative functional connectivity between seed regions and significant clusters (Fig 1B–1D).

### ROI-to-ROI analysis

ROI-ROI functional connectivity was compared between FMS patients and healthy control group using two-sided independent *t*-test analysis implemented in the CONN toolbox. There were no group differences in functional connectivity evident between any of the allocated DMN seed ROIs (P>0.05).

### Correlation analysis

**Fig 2:**
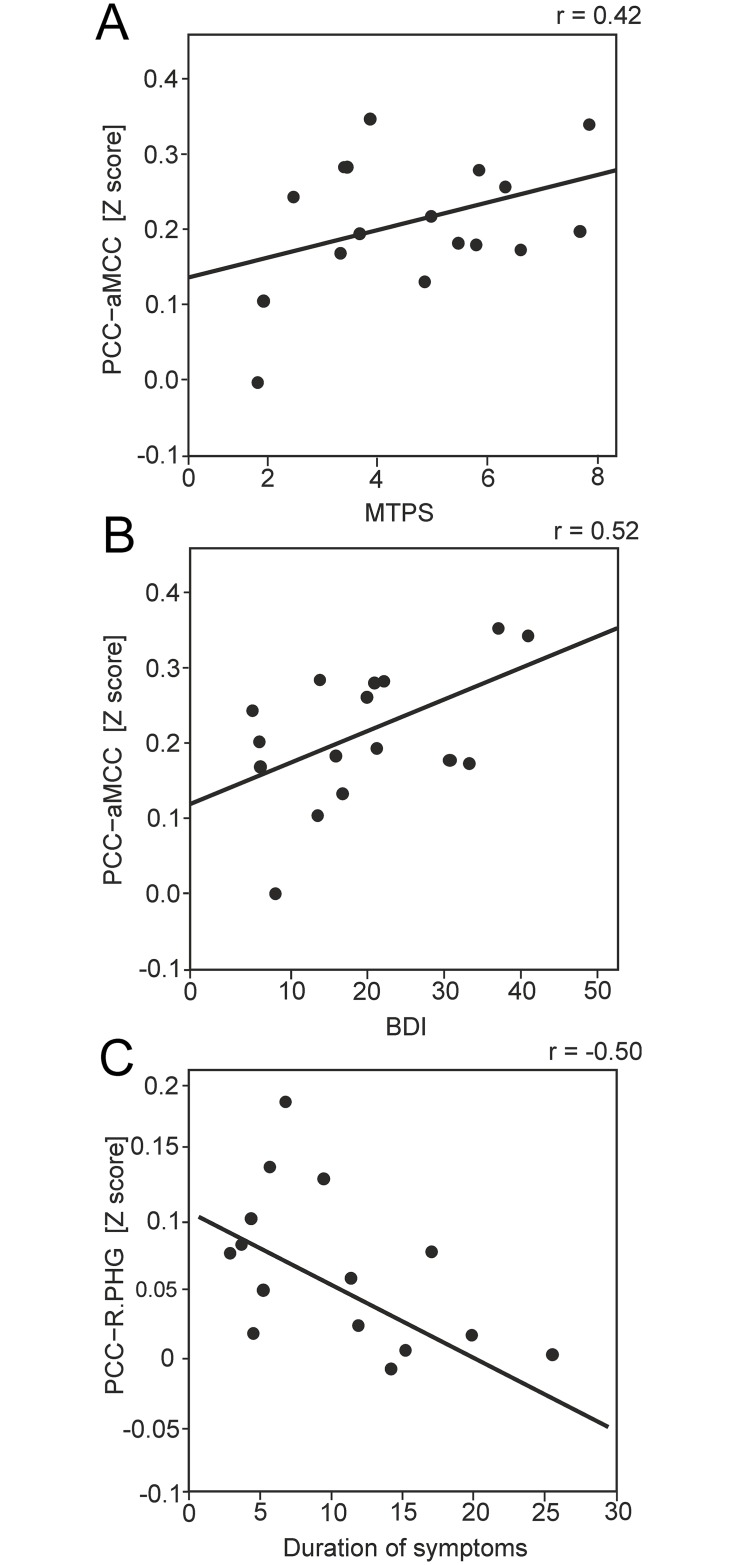
Correlation analysis. **A.** Scatter plot showing MTPS scores and Fischer transformed connectivity correlation coefficients representing relative functional connectivity between the aMCC and PCC and in FMS patient group. The linear regression line is also shown. **B.** Scatter plot showing BDI scores and relative functional connectivity between the aMCC and PCC in FMS patient group. **C.** Scatter plot showing duration of symptoms (in years) and relative functional connectivity between PCC and right parahippocampal gyrus in FMS patient group.

Pearson’s correlation analysis was performed between functional connectivity coefficients and clinical measures in the FMS patient group. The correlation coefficients for functional connectivity between PCC and aMCC, which demonstrated augmented connectivity in FMS, positively correlated with clinical measures including MTPS (r(14) = 0.43, P = 0.049) and BDI scores (r(14) = 0.52, P = 0.019), suggesting that the increased connectivity between these regions in FMS patients may relate to pain and affective symptoms. Fig 2A and 2B shows the scatterplots of correlation coefficients representing relative functional connectivity between PCC−aMCC and MTPS/BDI scores respectively. The connectivity between the cluster located in the right parahippocampal gyrus and the PCC seed, which demonstrated reduced connectivity in FMS patients relative to healthy control group, negatively correlated with the duration of symptoms in FMS patients (r = −0.50, p = 0.049). The degree of disruption to functional connectivity between PCC and parahippocampal gyrus was associated with longer symptom duration but not with other clinical measures relating to pain or affective disturbance. Fig 2C shows the scatterplot of correlation coefficients representing relative functional connectivity between PCC and parahippocampal gyrus and duration of symptoms in the FMS patient group.

## Discussion

FMS patients demonstrated increased functional connectivity between the DMN seed in the PCC and the aMCC which corresponds closely with the primary loci of anatomical brain alterations reported in a recent meta-analysis of studies of FMS patients [35]. Furthermore, the degree of augmented connectivity seen in patients positively correlated with widespread tenderness indexed by MTPS scores, and depression scores as measured by the BDI questionnaire, which suggests that increased connectivity between these regions could have relevance for the development and/or maintenance of predominant FMS symptoms. According to a recent meta-analyses, the aMCC is amongst the most commonly seen pain processing regions in both clinical and experimental pain neuroimaging studies [36–38]. This region has also been shown to play an important role in processing of tonic ongoing pain [39], and recent evidence points towards the aMCC sub-serving the relationship between pain and action selection [40–42], or approach/avoidance functions relevant for pain and decision-making (reviewed in [38]). Therefore, augmented connectivity between PCC and aMCC in FMS patients during rest may reflect the additional burden of decision-making and processing of appropriate action responses in the presence of chronic pain. The PCC in the DMN also showed reduced connectivity with right inferior temporal gyrus in FMS patients. This finding corresponds with reduced connectivity seen between these regions identified in patients with diabetic neuropathy which was proposed to indicate a reorganisation of resting brain networks contributing to spontaneous pain [43].

Reduced functional connectivity between the DMN seed in PCC and the right parahippocampal gyrus was also evident in the FMS patient group. The degree of disruption to connectivity correlated with the duration of symptoms in FMS patients but not tenderness scores or affective disturbance. This correlation is potentially indicative of a time-dependent alteration, although this cannot be specifically attributed to FMS symptoms. The right IPL structure in the DMN demonstrated increased connectivity with the right hippocampal formation in FMS patients relative to the healthy control group. Morphological changes in the hippocampal formation have previously been identified in FMS [44,45], and an animal model previously linked hippocampal neurogenesis to various chronic pain syndromes [46].

Augmented connectivity with the hippocampal formation was recently identified in long-term, but not early-stage, chronic back pain patients, and this reorganisation could represent the transition from acute to chronic pain [4]. A comparison of early and late stage chronic back pain patients revealed that long-term chronic pain caused a shift towards augmented activation patterns in brain regions related to cognitive-affective processing (including hippocampal formation), whereas early-stage pain was linked to activations in accepted pain processing regions [47]. Apkarian et al. [48] recently proposed that the development and maintenance of chronic pain could be contingent on the interaction between hippocampal learning mechanisms and nociceptive signals. As such the augmented hippocampal connectivity seen in the present study could be of relevance for the development of chronic symptoms in FMS. The hippocampal formation was also previously included as an additional structure of the DMN [49,50], and functional connectivity analysis of this seed region demonstrates strong correlations with DMN activity [51]. This overlap points to a link between DMN and networks underlying cognitive function and memory [49,52] which are often impaired in FMS [53–55].

FMS patients also demonstrated increased functional connectivity between the left IPL structure in the DMN and the left superior parietal lobule. As this region is adjacent to the parietal activation patterns seen in the DMN, the augmented connectivity is likely to reflect an expansion of the DMN to incorporate this area in FMS patients, perhaps as a result of ongoing pain. Hyperperfusion was previously seen in the left superior parietal lobule in FMS patients at rest [56] and similar augmented DMN connectivity with this region predicted clinical pain following movements in chronic back pain patients [19].

Due to the heterogeneity of FMS populations, it was proposed that predominant symptom profiles within a patient group can influence findings [57], which may relate to the pattern of connectivity differences in the present study, and indeed the diversity of findings in the literature. Our patient population were predominantly taking minimal medications suitable for a short withdrawal or not taking medication at all [27], which could affect the severity or symptom profile of patients taking part. Therefore, patient heterogeneity can be considered as a limitation of this method for investigation of FMS, although this would also suggest that functional connectivity could be sensitive enough to evaluate specific symptom profiles which would be advantageous. Future studies should consider sub-grouping patients based on symptom profiles, or longitudinal studies could relate the prevalence or dynamics of particular symptoms to specific functional connectivity alterations. As with all cross-sectional research, it is not possible to infer causality for any of the alterations seen in the present study, and future longitudinal research is needed to consider the direction and progression of the relationship between resting-state alterations and symptoms in FMS. Furthermore, although we utilised MTPS examinations to consider tenderness on the day of testing, a specific measure of clinical pain levels, e.g., McGill Pain Questionnaire [58] would also represent a beneficial addition in future.

Our findings suggest that FMS alters DMN connectivity with brain regions such as aMCC, hippocampal formation and inferior temporal gyrus which have implications for pain and developing chronicity, action selection, decision making, and affective/cognitive disturbances in FMS. The locations of structures exhibiting altered functional connectivity were either anatomically or functionally linked to the DMN suggesting a re-organisation of resting-state networks in FMS patients. These alterations could reflect predisposing central factors for the development of FMS, or alternatively, the consequences of ongoing symptoms on resting brain activity. Furthermore, connectivity increases between PCC and aMCC were correlated with clinical measures in patients, whereas reduced connectivity with parahippocampal gyrus was associated with longer symptom duration in FMS patients which may reflect ongoing time-dependent reorganisation or an indication of developing chronicity. Our results indicate that FMS symptoms are likely to influence functional connectivity with the DMN, and the specific nature of connectivity alterations could be of importance for understanding specific mechanisms underlying FMS. However, the pattern of alterations may vary as a result of the heterogeneous symptom profile of the patient cohort or fluctuations in levels of ongoing pain and other symptoms.
